# Phlegmasia cerulea dolens after endovenous cyanoacrylate closure of incompetent small saphenous vein

**DOI:** 10.1093/jscr/rjae363

**Published:** 2024-05-29

**Authors:** Yohei Kawatani, Hirofumi Saitoh, Takaki Hori

**Affiliations:** Cardiovascular Surgery, Kamagya-General Hospital, 929-6, Hatsutomi, Kamagaha-Shi, Chiba-Ken 273-0121, Japan; Internal Medicine, Kamagya-General Hospital, 929-6, Hatsutomi, Kamagaha-Shi, Chiba-Ken 273-0121, Japan; Cardiovascular Surgery, Kamagya-General Hospital, 929-6, Hatsutomi, Kamagaha-Shi, Chiba-Ken 273-0121, Japan

**Keywords:** cyanoacrylate, phlegmasia cerulea dolens, deep venous thrombosis, incompetent saphenous vein

## Abstract

A previously healthy 70-year-old woman underwent cyanoacrylate closure of an incompetent left small saphenous vein. Six days later, grade 2 treatment-induced thrombosis occurred at the sapheno-popliteal junction. Three days later, the patient presented with pale, cold pain in the left lower extremity. Diffuse thrombosis of the left lower extremity involved the small and great saphenous and deep veins. The patient was admitted and immediately administered heparin with anticoagulant factors, with symptoms began resolving 1 d later. The superficial vein thrombi were resolved. Although a deep venous thrombus remained, symptoms disappeared, and the patient was discharged.

## Introduction

Venous thrombosis is a well-known complication of endovenous ablation for treating saphenous vein insufficiency [1–3]. Endovenous ablation-induced thrombosis is also known as endovenous heat-induced thrombosis (EHIT) or endovenous glue-induced thrombosis (EGIT). However, most of these complications are mild [4]. In this study, we encountered a case of severe manifestation of venous thrombosis presenting as phlegmasia cerulea dolens (PCD) following endovenous cyanoacrylate closure (CAC) for treating small saphenous vein insufficiency.

## Case presentation

A 75-year-old woman contacted the emergency medical services 9 d after undergoing CAC for bilateral small saphenous vein insufficiency and reported acute swelling, pain, and paleness in the left lower extremity.

The patient was healthy, except for presenting with hypertension and dyslipidemia. The patient had no history of deep venous thrombosis (DVT) or thrombotic predisposition.

Operative procedures were performed using a CAC device (Venaseal, Medotoronic, Dublin, Ireland), according to the manufacturer’s instructions. Ultrasound examination at the end of surgery confirmed the presence of a precise occlusion, which was 2 cm distally to the small saphenous–popliteal vein junction. Examination 6 d after surgery revealed a grade 2 EGIT, which was observed without medication.

**Figure 1:**
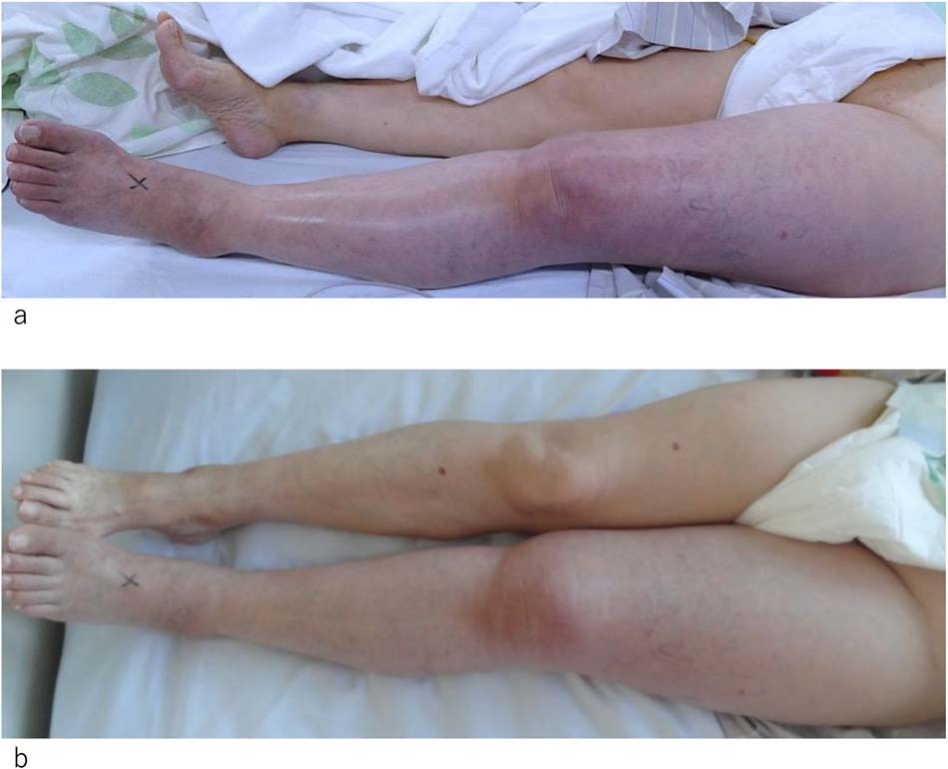
Photograph of the affected leg. (a) At the day of admission. The left leg appears pale and swollen. The superficial veins are occluded. (b) One day after admission. The color of the skin and occlusion of the superficial vein appears improved. Swelling of the leg is still observed.

**Figure 2:**
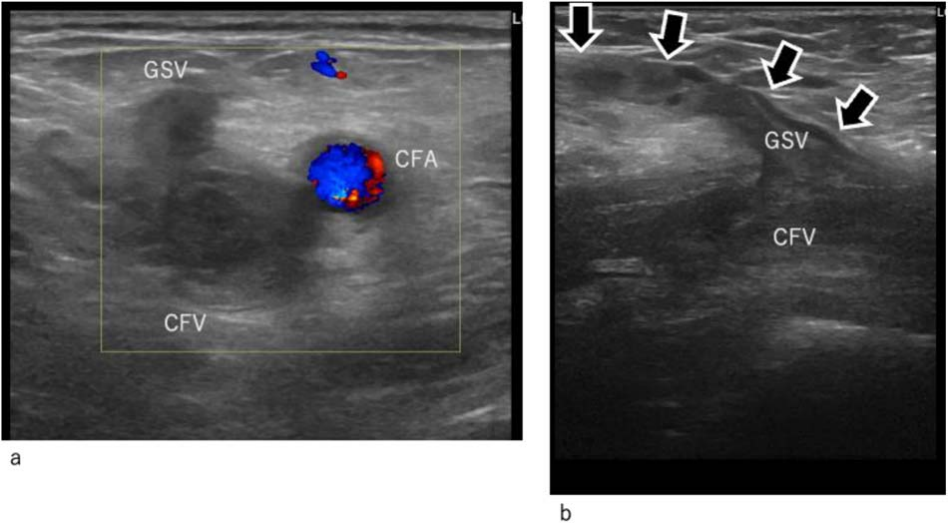
Duplex ultrasound image at arrival. (a) Transverse image. (b) Longitudinal image. The common femoral and GSV (arrow mark) are occluded by thrombi. CFA, common femoral artery; CFV, common femoral vein; GSV, great saphenous vein.

On postoperative Day 9, the patient experienced pale, cold pain in the left lower extremity (Fig. 1a). The symptoms progressed rapidly and at 4 h after their onset, the patient could not walk because of pain and paralysis in the left lower extremity. Ultrasonography revealed total occlusion of the left deep veins from the common iliac to the popliteal vein. Moreover, occlusion was also observed in superficial veins, including the great saphenous vein (GSV) and its branches (Fig. 2a and b). Arterial ultrasound examination showed no thrombus or occluded lesion but impaired pulsatile flow in the left lower extremity. Blood tests revealed very high levels of D-dimer (25 ng/mL) and fibrinin/fibrinogen degradation products (100 μg/mL).

Systemic administration of heparin was initiated in the emergency room and continued in the high-care unit. Despite this, the activated clotting time was ~130 s. Suspecting a fibrinolytic protein shortage, antithrombin III agents and plasma transfusion were administered.

**Figure 3:**
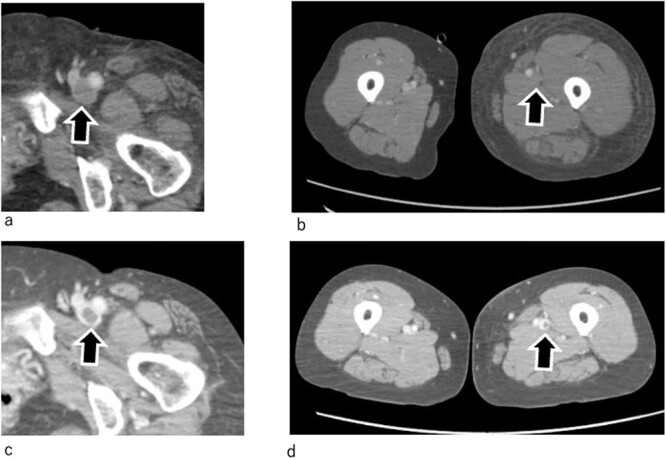
Enhanced CT. (a, b) One day after admission. The thrombus in the GSV has disappeared. However, thrombi are still observed in the deep veins, which remain totally occluded. (c, d) On Day 7. The thrombi in the deep veins have shrunk, and the veins have recanalized. Arrow marks indicate femoral veins.

By Day 2 of hospitalization, the pale skin of the patient and pain started to improve, and she was able to move her left lower extremity (Fig. 1b). Enhanced computed tomography (CT) revealed the resolution of the thrombus in the GSV; however, the deep veins remained completely occluded by thrombi (Fig. 3a and b). An ultrasound arteriogram revealed normal arterial blood flow.

On Day 9 of hospitalization, the leg swelling resolved. Contrast-enhanced CT revealed partial recovery of blood flow in the deep veins (Fig. 3c and d). In addition, the swelling of the left lower extremity resolved (Fig. 3b and d). Following this, heparin was changed to the oral anticoagulant apixaban (10 mg/d).

Although a deep venous thrombus remained in the common iliac vein to the superficial femoral vein, the left lower extremity swelling resolved. The patient was discharged asymptomatic.

## Discussion

EGITs are common [1–3]; however, most EGITs are not associated with clot extension or migration [2, 4]. In patients with grade 1 or 2 EGITs, administration of anticoagulant or antiplatelet agents is considered unnecessary because these typically resolve on their own, as previously observed [2, 3].

In our case, only grade 2 EGIT was noted on postoperative Day 6; however, 3 d later, the patient experienced the most severe manifestation of venous thrombosis. The thrombus diffused into the iliac, femoral, popliteal, and GSV.

Gressenberger et al. reported a case of DVT after CAC due to glue extension into the common iliac vein [5]. In the case, the thrombus extended from the GSV to the common femoral vein near the treated area. In contrast, our patient experienced broad thrombosis. Anatomically, the pelvic veins and GSV are not near the treated vein. Based on records and findings, the etiology was assumed to be thrombus extension caused by EGIT in the small saphenous–popliteal vein. However, this hypothesis remained unverified as simple thrombus extension affects veins far from the treated region. There is a possibility that, as Parsi et al. warned, systemic thromboembolic events may occur after CAC [1].

PCD is a rare and extreme manifestation of deep and superficial venous thrombosis that can lead to limb loss and ultimately death [6]. Extreme hypertension in veins can lead to impaired arterial blood flow, which can lead to venous gangrene if untreated [7]. Triggering factors for PCD include surgery, femoral vein catheterization, pregnancy, leg stasis, hypercoagulability, and vascular wall injury [8, 9]. A universal treatment approach for PCD has not yet been established. Currently, the initial treatment for PCD is bedrest, while intensive care includes fluid resuscitation and systemic heparin administration [10, 11]. Systemic administration of thrombolytic agents [12] or catheter-directed thrombolysis are some of the options for treating DVT [13]. Surgical resection of the thrombus is also an alternative option [14].

Post-CAC PCD is an extremely rare complication. In this case, heparin was administered along with antithrombin III agents and plasma transfusion to correct the fibrinolytic system. The superficial veins were recanalized. Consequently, the leg and life of the patient were saved. The successful outcome was attributed to the early administration of heparin after onset, which was ~4 h. The critical point of resolution in PCD is the resolution of the superficial acute thrombus. This improves venous blood flow via the superficial venous network, which resolves the low tissue perfusion caused by the occlusion. In the present case, although the deep vein occlusion persisted, the symptoms of the patient and physical findings improved.

Theoretically, PCD is established when thrombosis extends to the collateral veins, with massive fluid sequestration, greater edema, and low arterial perfusion [15]. Therefore, we assumed that thrombus formation in the collateral veins is a critical treatment goal in PCD. In this case, thrombus disappearance and symptom improvement occurred simultaneously. Despite the persisting DVT, the limb and life of the patient were rescued. Therefore, we did not perform additional radical treatment for DVT other than heparin administration followed by oral anticoagulants.

In conclusion, this case demonstrated that the critical point in PCD treatment after CAC is the immediate initiation of anticoagulation therapy to resolve thrombosis in the collateral veins.

## References

[1] Parsi K , Zhang L, Whiteley MS, et al. 899 serious adverse events including 13 deaths, 7 strokes, 211 thromboembolic events, and 482 immune reactions: the untold story of cyanoacrylate adhesive closure. Phlebology 2023;30:2683555231211086. 10.1177/02683555231211086 37902099

[2] Pappas JN , Pappas PJ, Lakhanpal S, et al. Natural history and role of anticoagulation in the management of endovenous glue-induced thrombus. J Vasc Surg Venous Lymphat Disord 2023;11:938–45. 10.1016/j.jvsv.2023.03.021. 37353153

[3] Sermsathanasawadi N , Pruekprasert K, Prapassaro T, et al. Thrombus extension after cyanoacrylate closure of incompetent saphenous veins. Int Angiol 2022;41:143–8. 10.23736/S0392-9590.22.04768-X. 35005874

[4] Cho S , Gibson K, Lee SH, et al. Incidence, classification, and risk factors of endovenous glue-induced thrombosis after cyanoacrylate closure of the incompetent saphenous vein. J Vasc Surg Venous Lymphat Disord 2020;8:991–8. 10.1016/j.jvsv.2020.01.009. 32179036

[5] Gressenberger P , Portugaller RH, Gütl K, et al. Persistent iliac vein thrombosis after cyanoacrylate closure of the great saphenous vein. J Dtsch Dermatol Ges 2020;18:1322–4. 10.1111/ddg.14258_g. 32881323

[6] Barrack RL , Butler RA. Avoidance and management of neurovascular injuries in total hip arthroplasty. Instr Course Lect 2003;52:267–74. 12690854

[7] Perkins JM , Magee TR, Galland RB. Phlegmasia caerulea dolens and venous gangrene. Br J Surg 1996;83:19–23. 10.1002/bjs.1800830106. 8653352

[8] Patel NH , Plorde JJ, Meissner M. Catheter-directed thrombolysis in the treatment of phlegmasia cerulea dolens. Ann Vasc Surg 1998;12:471–5. 10.1007/s100169900187. 9732427

[9] Warkentin TE , Elavathil LJ, Hayward CP, et al. The pathogenesis of venous limb gangrene associated with heparin-induced thrombocytopenia. Ann Intern Med 1997;127:804–12. 10.7326/0003-4819-127-9-199711010-00005. 9382401

[10] Mesfin A , Lum YW, Nayfeh T, et al. Compartment syndrome in patients with massive venous thrombosis after inferior vena cava filter placement. Orthopedics 2011;34:229. 10.3928/01477447-20110124-23. 21410121

[11] Brockman SK , Vasko JS. Phlegmasia cerulea dolens. Surg Gynecol Obstet 1965;121:1347–56. 5322039

[12] Chinsakchai K , Ten Duis K, Moll FL, et al. Trends in management of phlegmasia cerulea dolens. Vasc Endovascular Surg 2011;45:5–14. 10.1177/1538574410388309. 21193462

[13] Goldhaber SZ , Magnuson EA, Chinnakondepalli KM, et al. Catheter-directed thrombolysis for deep vein thrombosis: 2021 update. Vasc Med 2021;26:662–9. 10.1177/1358863X211042930. 34606385 PMC9009765

[14] Ruben-Castillo C , Cuen-Ojeda C, Lopez-Peña G, et al. Surgical intervention for phlegmasia cerulea dolens in a 61-year-old cancer patient. Tex Heart Inst J 2022;49:e207400. 10.14503/THIJ-20-7400. 35099561 PMC8884283

[15] Tan M , Sadek M, Kabnick L, et al. Management of endothermal heat-induced thrombosis. Phlebology 2023;39:214–7. 10.1177/02683555231219549. 38047878 PMC10938481

